# Retraction: Fabrication of divalent ion substituted hydroxyapatite/gelatin nanocomposite coating on electron beam treated titanium: mechanical, anticorrosive, antibacterial and bioactive evaluations

**DOI:** 10.1039/d3ra90025h

**Published:** 2023-03-29

**Authors:** A. Karthika, L. Kavitha, M. Surendiran, S. Kannan, D. Gopi

**Affiliations:** a Department of Chemistry, Periyar University Salem 636011 India dhanaraj_gopi@yahoo.com +91 427 2345124 +91 427 2345766; b Department of Physics, School of Basic and Applied Sciences, Central University of Tamilnadu Thiruvarur 610101 India; c Department of Zoology, School of Life Sciences, Periyar University Salem-636 011 India; d Centre for Nanoscience and Nanotechnology, Periyar University Salem 636011 India

## Abstract

Retraction of ‘Fabrication of divalent ion substituted hydroxyapatite/gelatin nanocomposite coating on electron beam treated titanium: mechanical, anticorrosive, antibacterial and bioactive evaluations’ by A. Karthika *et al.*, *RSC Adv.*, 2015, **5**, 47341–47352, https://doi.org/10.1039/C5RA05624A.

The Royal Society of Chemistry, with the agreement of the named authors, hereby wholly retracts this *RSC Advances* article due to concerns with the reliability of the data in the published article.

Repeating fragments can be observed within the 50 μg mL^−1^ panel in Fig. 7a, and in the control panel in Fig. 7b. An identical fragment is also duplicated in the 25 μg mL^−1^ and 50 μg mL^−1^ panels in Fig. 7b.

Given the significance of the concerns about the validity of the data, the findings presented in this paper are not reliable.

M. Surendiran was contacted but did not respond. S. Kannan responded but did not confirm whether they agreed to retract the article.

Signed: A. Karthika, L. Kavitha and D. Gopi

Date: 16th March 2023

Retraction endorsed by Laura Fisher, Executive Editor, *RSC Advances*

## Supplementary Material

